# 4D flow MRI demonstrates altered aortic hemodynamics in patients with right-left and right-noncoronary bicuspid aortic valve fusion patterns

**DOI:** 10.1186/1532-429X-15-S1-M8

**Published:** 2013-01-30

**Authors:** Riti J Mahadevia, Susanne Schnell, Pegah Entezari, Daniel Rinewalt, Preeti Kansal, SC Malaisrie, Patrick McCarthy, Jeremy Collins, James Carr, Alex J Barker, Michael Markl

**Affiliations:** 1 Northwestern University, Chicago, IL, USA; 2 Rush University Medical Center, Chicago, IL, USA

## Background

Altered aortic hemodynamics associated with bicuspid aortic valve (BAV) have been implicated in the development of aortopathy in patients with congenitally altered valves[1]. Most studies have focused on the most common "right-left" bicuspid aortic valve (RL-BAV) fusion pattern [2]. This study assessed the effects of valve morphology on aortic 3D blood flow in cohorts with the most common RL-BAV fusion, less common right-noncoronary (RNC-BAV), and normal trileaflet valves.

## Methods

**Figure 1:**
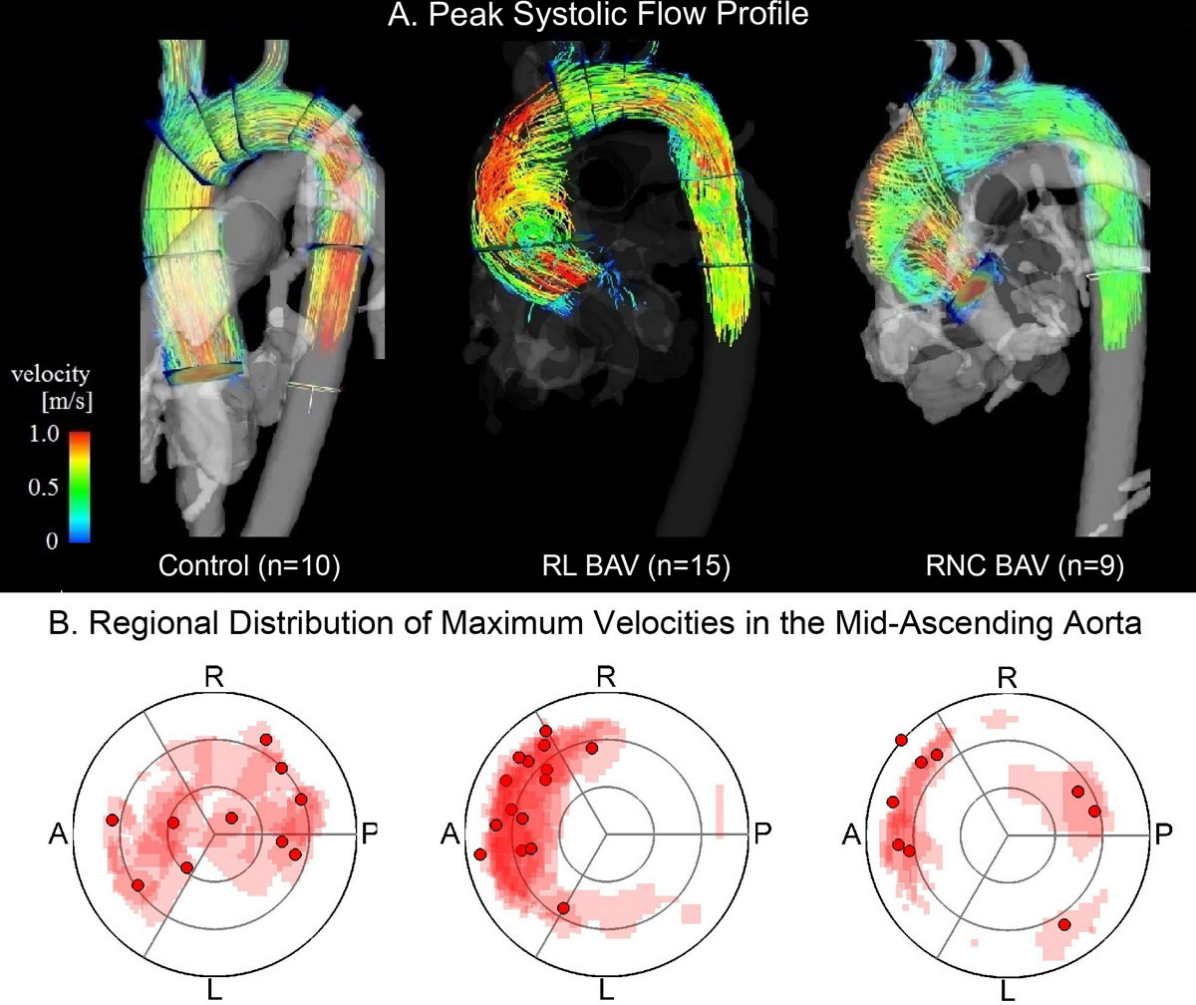
(A.) 4D flow MRI visualization of peak systolic flow using 3D pathlines (color-coded for velocity gradient) demonstrated asymmetric helical flow in RL- and RNC- BAV patients compared to more uniformly distributed flow in trileaflet controls. (B.) Locations of the top 15% of systolic velocities (mapped across a 2D analysis plane in the mid-ascending aorta) were centrally distributed in controls and concentrated towards the outer aortic wall in BAV patients. RNC-BAV subjects showed more variation in distribution compared to RL-BAV, whose flow profile was more consistently directed towards the right-anterior wall.

4D flow MRI was performed on 1.5T and 3T scanners with full thoracic aorta volumetric coverage (spatial resolution=2.9x2.1x2.5mm, temporal resolution=38.4ms) in 24 BAV patients (n=15 RL-BAV, age=47.2±11.7, mid-ascending aortic diameter=4±0.7 cm; n=9 RNC-BAV, age=47.4±10.6, diameter=3.9±0.5cm) and n=10 controls with trileaflet valves (age=28.4±2.4, diameter=2.7±0.3 cm). Time-resolved 3D pathlines (EnSight, CEI, USA) were used to visualize and assess flow uniformity and presence of helix flow. A 2D analysis plane was manually positioned in the mid-ascending aorta to quantify systolic peak velocities, retrograde fraction, net flow, valve outflow angle, and position of peak systolic velocities (location of the top 15% of velocities; figure 1).

## Results

4D flow MRI revealed systolic helical flow with asymmetric peak velocities in all BAV patients compared to more uniformly distributed flow in controls (figure 1A). Consistent with cohesive streamlines, controls had centrally distributed peak systolic velocities (figure 1B, left). In contrast, peak velocities were concentrated towards the outer aortic walls in BAV patients, consistent with enhanced flow asymmetry and jet flow patterns in BAV. While RNC-BAV had more eccentric distributions (n=6 focused in the anterior aorta, n=3 posterior), systolic flow profiles in RL-BAV had highly consistent flow jets directed at the right-anterior wall in all n=15 subjects. As summarized in table 1, BAV was associated with significantly increased (p<0.05) systolic peak velocities (RL-BAV: 1.96±0.7m/s, RNC-BAV: 2.11±0.9m/s, control: 1.12±0.2m/s) and flow angle (RL-BAV: 33.15±20.2°; RNC-BAV: 25.8±9.5°, control: 9.54±4.7°).

**Table 1:** 

	Subject Characteristics			p-value (student's t-test)			p-value
	
	Control	RL-BAV	RNC-BAV	(Control v. RL)	(Control v. RNC)	(RL v. RNC)	ANOVA
n	10	15	9	-	-	-	-
Age	28.4±2.4	47.2±11.7	47.4±10.6	<0.001	<0.001	0.937	<0.001
Male	6	9	6	-	-	-	-
Aortic Insufficiency (moderate-severe)	0	2	1	-	-	-	-
Aortic Stenosis (moderate-severe)	0	1	1	-	-	-	-
Diameter (cm) Mid Ascending Aorta	2.7±0.3	4.0±0.7	3.9±0.5	<0.001	0.001	0.634	<0.001
Peak Velocity (m/s) Mid Ascending Aorta	1.12±0.2	1.96±0.7	2.11±0.9	0.001	0.006	0.956	0.006
Peak Velocity (m/s) Mid Aortic Arch	1.05±0.1	1.25±0.7	1.51±0.8	0.458	0.108	0.148	0.040
Peak Velocity (m/s) Mid Descending Aorta	1.29±0.2	1.05±0.3	1.33±0.6	0.005	0.750	0.158	0.080
Flow Angle(degrees)	9.54±4.7	30.20±13.4	30.74±22.9	<0.001	0.024	0.950	0.003

## Conclusions

The presence and type of BAV fusion was clearly associated with changes in systolic flow uniformity, peak systolic velocities, and flow angles. Flow jet patterns directed towards the aortic wall in BAV patients identify potential mechanisms for altered wall shear forces and aortopathy. Compared to RL-BAV, RNC-BAV showed more variable post-valve hemodynamics, indicating the potential for more complex and diverse outcomes in RNC-BAV subtypes. Future longitudinal studies are thus warranted to evaluate the impact of BAV valve morphology and altered hemodynamics on variability in aortopathy development.

## Funding

NIH R01HL115828; NUCATS Dixon Award

## References

[B1] Girdauskas Eur J Cardiothorac Surg, 2011 39 6 809 814 10.1016/j.ejcts.2011.01.001 21342769

[B2] Barker Markl Eur J Cardiothorac Surg, 2011 39 6 805 806 10.1016/j.ejcts.2011.01.006 21339071

